# Evaluation of a Phytogenic Compound with Minerals as a Possible Alternative to Ractopamine for Finishing Pigs

**DOI:** 10.3390/ani12182311

**Published:** 2022-09-06

**Authors:** Valéria dos Santos Moreira, Cesar Augusto Pospissil Garbossa, Eduarda Buck Bernardes Guimarães, Welinton Yoshio Hirai, Thiago Augusto da Cruz, Laya Kannan Silva Alves, Lucio Francelino Araujo

**Affiliations:** 1 Faculty of Zootechnics and Food Engineering, University of São Paulo, Pirassununga 13635-900, Brazil; 2 Faculty of Veterinary Medicine and Animal Science, University of São Paulo, Pirassununga 13635-900, Brazil; 3 Luiz de Queiroz Agricultural Faculty, University of São Paulo, Piracicaba 13418-900, Brazil; 4 School of Life Sciences, Pontifical Catholic University, Curitiba 80215-901, Brazil

**Keywords:** swine, performance, pork

## Abstract

**Simple Summary:**

The pork consumer market has restrictions on many products used in production, including ractopamine. Therefore, it is important to search for alternatives so that the animal continues to achieve maximum weight gain with a leaner carcass. We performed an evaluation comparing animals whose diet was supplemented with ractopamine, a diet with a phytogenic compound, and a diet without additives. Animals whose diet was supplemented with ractopamine performed better, and animals whose diet was supplemented with the herbal extract performed intermediately when compared with animals whose did not receive additives in their diet. The animals that were fed the phytogenic compound had improved the sensory attributes of the pork.

**Abstract:**

The objective of this work was to evaluate the effect of a phytogenic compound for pigs in the growing and finishing phases as a possible substitute for ractopamine. A total of 140 pigs with an average initial weight of 48.8 kg ± 5.9 kg were used, distributed in a randomized block design, in a 3 × 2 factorial scheme (control diet (CONT), diet with inclusion of 2.5 kg per ton of a phytogenic compound (PC), and diet with 10 ppm of ractopamine (RAC), and two sexes: gilts and barrows), distributed in eight pens per treatment. The performance parameters were measured, and, at the end of the experimental period, the animals were slaughtered for carcass characteristics and pork quality analysis. The animals consuming RAC showed a better feed conversion, 4% improvement in relation to the group with the PC (*p* < 0.05). For daily weight gain, the animals supplemented with the PC showed 4.46% lower gain compared to RAC, and 3% greater gain compared to the CONT (*p* < 0.05). The animals that consumed the PC showed 5.6% lower shear force of the pork (*p* < 0.05) in relation to the CONT group and 29% lower in relation to the RAC group. The TBARS value presented a significant difference (*p* < 0.05), the CONTT group was 29% higher than the RAC, and the PC was 15.5% higher than the RAC. For chroma, the pork of the RAC group was 14% lower than the CONTT group and 10.3% less than the PC. There was no significant difference for the carcass parameters. It was concluded that the pigs in the ractopamine group presented the best performance; however, the phytogenic compound can be used against ractopamine’s restriction because it improves daily weight gain and promotes a softer and less pale meat when compared with ractopamine.

## 1. Introduction

The growing demands of the consumer market for a safe, nutritious, and sensorially pleasant meat represent one of the main motivations for the improvement of the sector [1]. Allied to meat quality issues, alternatives are sought for an economic optimization in production.

Among the tools that can be used to increase lean meat in the carcass are genetics, nutrition, and the use of carcass modifiers [2].

In order to optimize production efficiency, ractopamine, a synthetic and beta-adrenergic additive is used to improve growth performance and carcass characteristics [3], which has the potential to increase the efficiency of muscle tissue growth [4].

However, the use of ractopamine generates discussion about the countereffects on pigs as the animals tend to be more susceptible to stress due to the effect on heart rate and catecholamine [5]. In a review carried out by Ritter et al. [6], the authors concluded that the product makes it difficult to handle pigs as it increases their aggressive behavior. Conversely, studies indicate that ractopamine has no impact on the general welfare status of the animals [7].

According to Onishchenko et al. [8], regarding the consumption of animals supplemented with ractopamine, there is a concern for human health due to the risk of cardiovascular disorders. In this sense, the controversies about the additive divide markets. More than 160 countries have banned the use of ractopamine, while other countries consider its use safe [9], following the Food and Agriculture Organization of the United Nations (FAO) guidelines, which adopts the maximum limits established by the Codex Alimentarius [10].

Nevertheless, with the objective of continuing to serve the main consumer markets, alternatives are being sought for the withdrawal of ractopamine without losses in performance and meat quality.

Among the possible alternatives are phytogenic additives such as flavonoids, alkaloids, and tannins, among others that have beneficial effects in intestinal health, antioxidant capacity, and digestibility. Due to these effects, these kinds of natural additives can be studied as tools to improve performance and pork quality.

Another option in order to improve pork quality is the utilization of some minerals, because they can act as a cofactor of enzymes or also reduce the stress in animals, such as manganese or magnesium, respectively.

The objective of this work was to study the effect of a commercial phytogenic compound with minerals or ractopamine for growing and finishing pigs on performance and meat quality.

## 2. Materials and Methods

All procedures involving the handling of animals were carried out under the approval of the Use of Animals Ethics Committee of the School of Zootechnics and Food Engineering of the University of São Paulo, with the approval number: CEUA 7390020421.

### 2.1. Additive

The phytogenic compound used was supplied by the company Idena Animal Nutrition, under the trade name Vali MP^®^. The formula is composed of cinnamaldehyde: 10 g/kg, magnesium oxide: 70 g/kg, and organic manganese: 10 g/kg.

### 2.2. Experimental Design and Formulas

The experiment was carried out in the experimental swine breeding unit at the University of São Paulo for 69 days. A total of 140 Danbred (gilts and barrows) pigs, with an average weight of 48.6 ± 5.9 kg and 97 days of age, were used. The design was a randomized block in a 3 × 2 factorial scheme (control diet, diet with inclusion of 2.5 kg of a phytogenic compound, and diet with 10 ppm of ractopamine (last 28 days of trial) and 2 sexes: gilts and barrows).

The housing took place in pens with three animals each, and in four pens two animals were housed in each. The supply of water and food was ad libitum. For performance analysis, the pens were considered an experimental unit with a total of eight pens per treatment.

The experimental diets consisted of commercial diets composed of different treatments, as follows: control diet (CONT), diet with inclusion of 2.5 kg per ton of feed of a phytogenic compound (PC), and diet with inclusion of ractopamine (RAC). The nutritional levels were adjusted throughout the experiment, meeting the nutritional requirements of the animals. Thus, the period was divided into growing (15 days), finishing 1 (26 days), and finishing 2 (28 days).

The animals in the RAC group consumed the same feed as the CONT group during the growth and finishing 1 phases. In the finishing 2 phase, the ractopamine additive with amino acid adjustments was included (Table 1). At the end of each phase, the animals were individually weighed, as well as the leftovers from the feeders.

### 2.3. Carcass Analysis

At the end of the experiment, the animals were fasted for 12 h and sent to slaughter in a commercial slaughterhouse (SISP). The slaughter took place through stunning via the bloodstream and subsequent bleeding in the great vessels. Subsequently, they were scalded, depilated, eviscerated, and the carcasses closed longitudinally and weighed. Based on the slaughter weight, carcass yield was calculated according to Bridi and Silva [1].

The variables backfat thickness, muscle depth, and percentage of lean meat were measured using a Hennessy GP4/GP7 probe, where an optical sensor perceives a light color, which corresponds to fat, in the same way it captures a dark color, which corresponds to the muscle fraction. The analysis took place at the height of the last rib, the same point where the pH measurement was performed 45 min after slaughter.

For carcass analysis, all animals were evaluated, and each was considered an experimental unit.

### 2.4. Meat Quality

The carcasses were stored in a cold chamber for 24 h, and after this period, five carcasses per treatment were selected of the animals with the highest slaughter weight of each treatment. A sample from the longissimus dorsi muscle was removed to measure pH, color, drip loss, cooking water loss, shear force, and TBARS.

Longissimus dorsi muscle samples were sectioned into a 2.5 cm thick subsample, which was used for evaluation of pH, color, cooking losses, and shear force analysis. Another 1.5 cm subsample was divided in half and used for analysis of lipid oxidation and drip loss.

Lipid oxidation samples were placed on polystyrene trays containing absorbent paper, wrapped in oxygen permeable cling film, and left for three days under simulated retrial exposure conditions (2 °C and 1000 lux lighting).

### 2.5. Color and pH

After obtaining the 2.5 cm sample, they were oxygenated for 30 min at 4 °C. Then, an objective evaluation of meat color was performed using the CIELab System [11], using a portable spectrophotometer, model CM2500d (Konica Minolta Brasil, São Paulo, Brazil) with illuminants and an observation angle of 10°. Final values of L* (luminosity), a* (red intensity), and b* (yellow intensity) of each sample were obtained through the average of the three measurements. Immediately afterwards, the pH of each sample was measured using a portable digital pH meter (Hanna Instrumentos—model HI99163, São Paulo, Brazil). In addition, the chroma values and hue angle were calculated according to Bridi and Silva [1].

### 2.6. Cooking Losses and Shear Force

After color and pH measurement, the samples were weighed and roasted in an industrial electric oven (Model F130/L—Fornos Elétricos Flecha de Ouro Ind. E Com. Ltda., São Paulo, Brazil) equipped with a thermostat at 170 °C. The internal temperature of the samples was monitored using individual thermometers. When they reached an internal temperature of 40 °C, the samples were turned over and remained in the over until reaching an internal temperature of 71 °C, as recommended by the American Meat Science Association [12].

The samples were kept at room temperature until they cooled to ±25 °C, when they were weighed again to determine cooking losses (cooking losses were expected as a percentage of the initial weight).

After that, the samples were wrapped in plastic film and placed in a refrigerator (4 to 6 °C) for 12 h, and then six to eight cylinders (1.27 cm in diameter) were removed from each sample, parallel to the fibers to determine the shear force using the TMS-PRO texture analyzer equipment (Food Technology Corporation, Sterling, VA, USA) coupled with a Warner-Bratzler shear device with a speed set at 200 mm/min (AMSA, 2015) [12]. The shear force of each sample was considered as the mean of the repetitions.

### 2.7. Drip Losses

Approximately 50 g of the subsamples was weighed, suspended in inflated bags, ensuring that the sample did not come into contact with the bag, and kept in refrigeration (4 °C) for 24 h as proposed by Honikel [13]. After 24 h, the samples were reweighed, and drip losses were expressed as a percentage of the initial weight.

### 2.8. Lipid Oxidation

At the end of the three days of simulated retail exposure, pH and color analysis were performed again, as described above, and then the samples were tested for quantification of thiobarbituric acid reactive substances (TBARS). For this analysis, 5 grams of samples were homogenized with thiobarbituric acid (TBA) and incubated in a water bath for 40 min at 100 °C, for the determination of thiobarbituric acid (TBARS), according to the methodology proposed by Vyncke [14] and modified by Sorensen and Jorgensen [15].

A standard curve with eight points was also prepared, using a tetraethoxypropane solution of known concentration to obtain the malondialdehyde concentration in the samples from the equation provided by the curve. Absorbance readings were taken at wavelengths of 532 nm and 600 nm. The results were expressed in μg malonaldehyde/kg sample.

### 2.9. Statistical Analysis

The SAS 9.4 software (SAS/STAT, SAS Institute Inc., Cary, NC, USA) was used for the analysis, in which all data were submitted to the Shapiro–Wilk test to confirm the normality of the residues.

Data were submitted to ANOVA, for the performance analysis, the pen was considered the experimental unit, and, for the carcass and meat quality analyses, each animal was considered the experimental unit. The model included the initial weight of the pigs as block, and diet and sex as fixed effects.

For the performance analyses, the following model was used:

y_kjl_ = m + b_j_ + A_k_ + B_l_ + (AB)_kl_ + e_klj_

where:

- Y is the response variable.
- m is the overall mean of the experiment.
- b_j_ is the block effect.
- A_k_ is the A factor, with k levels: sex effect with 2 levels.
- B_l_ is the B factor, with l levels: diet effect with 3 levels.
- (AB)_kl_ is the interaction between the factors.
- e_klj_ is the error associated with observation y_klj_.

For meat and carcass quality analysis, the model

y_klj_ = m + A_k_ + B_l_ + (AB)_kl_ + e_klj_

was used. Where:

- Y is the response variable.
- m is the overall mean of the experiment.
- A_k_ is the A factor, with k levels: sex effect with 2 levels.
- B_l_ is the B factor, with l levels: diet effect with 3 levels.
- (AB)_kl_ is the interaction between the factors.
- e_klj_ is the error associated with observation y_klj_.

If H = 0: all means are equal.

If H ≠ 0: at least one mean is different from the others, in this case, Tukey’s test was applied at a significance level of 5%.

## 3. Results

### 3.1. Animal Performance

Based on the results observed in Table 2, for all parameters evaluated, no interaction between diet and sexual status was observed.

In the phases in which only two rations were used, from growth to termination 1, there was no significant difference for performance parameters. In the finishing 2 phase, when ractopamine was included in the RAC treatment, the animals presented a feed conversion 4.5% lower in relation to the CONT group and 4.4% lower in relation to the PC group (*p* < 0.05).

RAC group pigs presented 4.4% greater ADG in relation to the PC, and the latter presented 3% greater ADG in relation to the CONT (*p* < 0.05).

For the zootechnical indices of the evaluated period from 1 to 69 days, there was no significant difference in the final weight of the animals (*p* > 0.05).

Feed efficiency was 4.4% higher in the RAC group when compared to the other treatments (*p* < 0.05), which did not differ statistically.

Regarding the females and castrated males, during the finishing 1 and finishing 2 phases, males had higher daily feed intake compared to females (*p* < 0.01), as well as greater daily weight gain.

The animals presented a statistically different final weight, where males weighed 6.7% more than females (*p* < 0.01) at the end of the experimental period.

When the parameters from 1 to 69 days were evaluated, males had a higher feed conversion compared to females, 6.22% (*p* < 0.01). Females consumed 14.6% less feed than males (*p* < 0.01) and had a 9% lower daily weight gain.

### 3.2. Carcass Characteristics

For the parameters evaluated (Table 3), there was no interaction between sex and diets. The animals consuming the different diets did not differ statistically from each other (*p* > 0.05). However, males presented a carcass with 0.93% higher yield compared to females (*p* < 0.01).

### 3.3. Meat Quality—24 h after Slaughter

Among the variables evaluated, there was no interaction between diet and sex regarding parameters related to meat quality (Table 4).

For the shear force, the animals of the PC group presented a value 5.6% lower in relation to the CONT group and 29% lower in relation to the RAC group (*p* < 0.05).

Regarding the observations on the different sexes, the female group presented meat 11% higher for a* and 7.53% lower for b* (*p* < 0.05).

### 3.4. Shelf Life of Meat—3 Days after Slaughter

In the observations made, there was no interaction between sex and treatment (Table 5).

In the results related to meat quality after 24 h of slaughter, there was a significant difference (*p* < 0.05) for the parameter related to red color intensity (a*) between the three treatments. The animals consuming the control diet showed a greater intensity of red in the meat, 9.4% above the animals of the PC group and 23% above the animals that consumed the RAC treatment.

The evaluated samples showed a significant difference (*p* < 0.05) for yellow intensity (b*): the CONT group presented 3.8% greater intensity of yellow in the meat compared to the PC group and 13.8% greater intensity of yellow when compared to the RAC group.

For chroma value, there was a significant difference (*p* < 0.05) between the RAC group and the other treatments. The RAC group showed 17% less saturation of meat color than CONT group and 11.4% less saturation when compared to PC group.

In the samples analyzed three days after storage, the CONT group presented a meat with a 4% higher a* index in relation to the PC group and 15% higher in relation to the RAC group.

Regarding the b* index, the animals in the CONT group showed 3% greater intensity of yellow in relation to the PC group and 13% in relation to the RAC group (*p* < 0.05).

For chroma value, the RAC treatment group showed 18.67% less saturation against CONT (*p* < 0.05) and revealed a result 10.5% less than PC group.

For the TBARS values, the animals in the RAC group showed a lower value compared to the PC group, representing less than 15% in relation to it. Regarding the animals in the CONT group, the value was 29% higher than in the RAC group. The three treatments differed from each other statistically (*p* < 0.05).

For shelf-life parameters evaluated on sex status, there was significant difference (*p* < 0.05) in color a*, b*, and chroma value for samples analyzed 24 h after slaughter. After three days of slaughter, there was no difference between treatments.

## 4. Discussion

### 4.1. Performance

According to Moody et al. [16], regarding ractopamine, the beta-agonist binding connects to specific receptors on muscle and adipose cells. In the former, protein synthesis increases, as the replacement of lean tissue requires less energy, and in the latter, biochemical signals decrease fat deposition. The result is a leaner animal that makes better use of the feed consumed.

Similar results were found by Elmes et al. [17], with better daily weight gain (*p* < 0.05) and higher feed efficiency (*p* < 0.01), with 10 ppm ractopamine supplementation.

Thus, it is expected that the animals consuming ractopamine present a better feed conversion and a carcass with lower fat content. Therefore, the results observed in this work of feed conversion agree with the information cited by the authors.

Regarding the daily weight gain of the PC compared to the CONT group, the product has a hypertrophic effect on the muscle [18], in addition to promoting a reduction in adipogenesis. Therefore, the product has the potential to promote lean growth in finishing pigs [19].

In a study conducted on broilers, Choi et al. [20] tested a commercial mixture of phytogenic compounds called Vali MP^®^ with different inclusions and the results showed that the inclusion of 0.125% of the commercial product increased daily weight gain (*p* < 0.05) when compared to the control group.

When testing phytogenic compounds on the performance of finishing pigs, Luo et al. [21], testing 80 mg of cinnamaldehyde in the animals’ diet, observed higher average daily gain (*p* < 0.05) compared to the control group. Likewise, Li et al. [22] determined the optimal inclusion of 0.04% of a pine extract which increased the daily weight gain of animals by 14.7% compared to an inclusion of 0.06% (*p* < 0.05). Korniewicz et al. [23] found higher final weight and daily weight gain (*p* < 0.05) of animals supplemented with 0.05% hop extract in a basal diet.

Studies carried out with different phytogenic compounds are similar to this work regarding the results of daily weight gain found, indicating that the use of phytogenic compounds can bring an improvement in performance for pigs supplemented with these additives.

Androgen hormones produced in the tests, such as testosterone, stimulate muscle growth and nitrogen and phosphorus retention, adding to that changes the distribution of nutrients from subcutaneous fat synthesis to other parts of the body, improving feed conversion and enhancing performance [24].

Pigs that undergo surgical castration have their sexual gonads removed, thus, according to Bridi [25], castrated males are more precocious to deposit body fat than females. Therefore, this condition reflects in a worse feed conversion and feed consumption in relation to females [26].

### 4.2. Carcass Characteristics

In the variables related to the carcass trait, the animals did not show significant differences between the parameters of backfat thickness, muscle depth, and carcass yield. Agostini et al. [7] worked with two different levels of ractopamine and found no significant difference for backfat thickness and muscle depth; however, the authors observed a quadratic response for carcass yield.

Dávila-Ramírez et al. [27] carried out a study in which the animals were supplemented with two different inclusions of a plant extract and, after analyzing the carcasses, found no significant difference for backfat thickness, muscle depth, and percentage of lean meat.

### 4.3. Meat Quality—24 h after Slaughter and 3 Days of Storage

The results show that, both in the RAC and PC groups, the initial pH is not affected 24 h after slaughter. Among the factors that can contribute to a higher or lower pH are preslaughter stress, prolonged fasting, or animals with high muscle glycogen content [25]. Based on the results, it is observed that the treatments did not affect the mentioned parameters.

Leonardo [28] did not observe an effect of ractopamine on initial and final pH on the animal carcass, nor did he observe any difference between sex. In a study carried out by Oliveira [29], animals that consumed 10 ppm of ractopamine for 28 days showed no difference in initial pH; males consuming ractopamine had a lower pH compared to females after 24 h.

Hanczakowska et al. [30] found no significant difference in pigs supplemented with herbal extract at initial pH and staining after 24 h.

Pork with a lower a* value is less red, which is not interesting for the consumers’ acceptance [31]. Alternatively, lower b* values may be related to greater oxidative stability of intramuscular fat [32]. Regarding chroma value, the RAC group meat presented a paler color than other treatments within 24 h and three days after slaughter. The results suggest a reduction in concentration of oxymyoglobin resulting in lighter colored meat [33] in the RAC group. The results are similar to those found by Lima [34] and Stella [35] with ractopamine supplemented for pigs.

As for the reduction of lipid oxidation, Hanczakowska et al. [30] tested an extract composed of artichoke, celery, beetroot, onion, garlic, spinach, avocado, oatmeal, and parsley in pigs. TBARS analysis was performed 24 h after slaughter, and there was no significant difference. The samples were stored for 5 months and, after this period, evaluated again; the animals that consumed the plant extract had a lower value (*p* < 0.05) of TBARS, indicating a lower degree of oxidation.

Polyphenols (such as tannins and flavonoids) and essential oils are the main fractions and bioactive components in plants, with natural antioxidant properties [36]. According to Hanczakowska et al. [30], lipids are particularly susceptible to oxidation, and, therefore, it can be assumed that herbal extracts protect fatty acids from oxidative damage due to their antioxidant potential.

Another point that may contribute to improvement in coloration and reduction of oxidation is related to the effects on the reduction of lipid deposition related to the use of ractopamine.

The diets did not promote a significant difference in drip water loss and cooking strength, possibly because they did not affect the biochemical processes involved in the transformation of muscle into meat, nor promoted any type of stress to the animal in the preslaughter phase.

As Brustolini et al. [37], who tested 10 ppm of ractopamine in castrated and immunocastrated males, observed no significant difference was observed for water retention capacity and water loss during cooking in both treatments.

Likewise, Li et al. [22] evaluated different inclusions of a phytogenic extract, composed of flavonoid, phenolic compounds, alkaloid, tannin, terpene, and saponin, on the water retention capacity at the 3rd and 6th days after slaughter, and there was no significant difference between treatments.

The increase in the shear force of animals that consume ractopamine can be explained by the increase in the gene expression of calpastatin [28], which reinforces the results found in our work, whereupon the animals supplemented with ractopamine presented a greater shear force.

## 5. Conclusions

Based on the results, it was concluded that ractopamine pigs had the better performance than those with the other treatments, owing to the fact that it improves average daily gain and feed conversion. However, for markets that recommend the withdrawal of ractopamine from swine production, the phytogenic compound can be a substitute as it improves the average daily gain.

Furthermore, the phytogenic compound is not a synthetic product, so, there are no restrictions related to the use of this additive. Moreover, the phytogenic compound produces meat with desirable characteristics, such as high meat tenderness, an important sensory characteristic to offer the consumer. In this sense, this product has potential as a consumer friendly additive, and it can be preferred over ractopamine that presents a meat with greater shear force.

## Figures and Tables

**Table 1:** Diets used in the evaluation: growth feed, finishing 1, finishing 2, and the addition of additives: phytogenic compound (PC) and ractopamine (RAC).

		Growing Feed	Growing Feed PC	Feed Finishing 1	Feed Finishing 1 PC	Feed Finishing 2	Feed Finishing 2 PC	Feed Finishing 2 RAC
Ingredients	Unit							
Ground corn	kg/ton	728	728	760	760	790	790	727.5
Soybean meal	kg/ton	237	237	213	213	187	187	237
Soybean oil	kg/ton	10	10	7	7	5	5	10
RAC ^1^	kg/ton							0.5
PC ^2^	kg/ton		2.5		2.5		2.5	
Px G ^3^	kg/ton	25						
Px F ^4^	kg/ton			20				
PX F II ^5^	kg/ton					18		
PX F-II RAC ^5^	kg/ton							25
Nutrients	Unit							
Moisture	%	11.98	11.98	12.08	12.08	12.14	12.14	11.98
Dry matter	%	86.72	86.72	86.78	86.78	86.74	86.74	86.67
Crude protein	%	17.00	17.00	16.00	16.00	15.00	15.00	17.00
Ethereal extract	%	4.01	4.01	3.82	3.82	3.71	3.71	4.01
Crude fiber	%	2.73	2.73	2.64	2.64	2.53	2.53	2.73
Calcium	%	0.57	0.57	0.49	0.49	0.44	0.44	0.57
Total phosphorus	%	0.40	0.40	0.36	0.36	0.34	0.34	0.40
Phosphorus disp	%	0.28	0.28	0.24	0.24	0.22	0.22	0.28
Metabolizable energy	kcal/kg	3350.00	3350.00	3350.00	3350.00	3350.00	3350.00	3350.00
Lysine dig	%	0.9270	0.9270	0.8100	0.8100	0.7000	0.7000	0.9270
Methionine dig	%	0.2905	0.2905	0.2400	0.2400	0.2241	0.2241	0.2905
Total Met + Cis	%	0.5470	0.5470	0.4866	0.4866	0.4599	0.4599	0.5470
Threonine dig	%	0.6030	0.6030	0.5425	0.5425	0.5103	0.5103	0.6030
Tryptophan dig	%	0.1850	0.1850	0.1600	0.1600	0.1413	0.1413	0.1850
Ractopamine	ppm							10.00

^1^ Ractopamine; ^2^ Phytogenic Compound; ^3^ PX growth: composition—Kaolin, Calcitic Limestone, Dicalcium Phosphate, Common Salt, DL-Methionine, L-Lysine, L-Threonine, L-Tryptophan, Vitamin A, Vitamin D3, Vitamin E, Vitamin K3, Vitamin B1, Vitamin B2, Vitamin B6, Vitamin B12, Nicotinic Acid, Calcium Pantothenate, Folic Acid, Biotin, Iron Sulfate, Copper Sulfate, Zinc Sulfate, Manganese Sulfate, Calcium Iodate, Sodium Slenite, Phytase, Xylanase + Betaa Glucanase, Bacitracin Methylene Disalicylate; ^4^ PX Finishing I: composition—Kaolin, Calcitic Limestone, Dicalcium Phosphate, Common Salt, DL-Methionine, L-Lysine, L-Trypyophan, Vitamin A, Vitamin D3, Vitamin E, Vitamin K3, Vitamin B1, Vitamin B2, Vitamin B6, Vitamin B12, Nicotinic Acid, Calcium Pantothenate, Zinc Sulfae, Manganese Sulfate, Calcium Iodate, Sodium Selenite, Phytase, Xylanase + Beta Glucanase, Bacitracin Methylene Disalicylate; ^5^ ePX Finishing II: composition—Kaolin, Calcitic Limestone, Dicalcium Phosphate, Common Salt, DL-Methionine, L-Lysine, L-Threonine, L-Thyptophan, Vitamin A, Vitamin D3, Vitamin E, Vitamin K3, Vitamin B1, Vitamin B2, Vitamin B6, Vitamin B12, Nicotinic Acid, Calcium Pantothenate, Folic Acid, Biotin, Iron Sulfate, Copper Sulfate, Zinc Sulfate, Manganese Sulfate, Calcium Iodate, Sodium Selenite, Phytase, Xylanase + Beta Glucanase, Bacitricin Methylene Disalicylate. Px Finishing II and Finishin II Rac had the same ingredients.

**Table 2:** Performance of pigs supplemented with different additives over the period (values expressed in kg).

							*p*-Value		
Variable	CONT	PC	RAC	Gilt	Barrow	SEM	Diet	Sex	Diet × Sex
n	16	16	16	24	24				
Growth—1 to 15 days									
^1^ ADFI	2.36	2.35	2.37	2.22 ^b^	2.50 ^a^	42.200	0.988	0.001	0.906
^2^ ADG	1.17	1.17	1.15	1.11 ^b^	1.22 ^a^	18.700	0.920	0.002	0.747
^3^ FC	2.03	2.035	2.05	2.02	2.05	0.028	0.898	0.600	0.719
^4^ FE	0.60	0.50	0.49	0.50	0.49	0.007	0.884	0.460	0.761
Weight 15 days	66.20	65.80	66.10	64.50	67.60	0.950	0.988	0.119	0.963
Finishing I—16 to 41 days									
ADFI	2.81	2.85	2.85	2.58 ^b^	3.09 ^a^	50.300	0.852	<0.001	0.749
ADG	1.03	1.09	1.09	1.00 ^b^	1.15 ^a^	16.700	0.135	<0.001	0.407
FC	2.71	2.63	2.62	2.61	2.70	0.030	0.366	0.115	0.143
FE	0.37	0.38	0.38	0.39	0.37	0.004	0.395	0.152	0.148
Weight 41 days	93.03	94.10	94.50	90.40 ^b^	97.40 ^a^	1.130	0.857	0.002	0.802
Finishing 2—42 to 69 days									
ADFI	2.96	3.06	3.07	2.80 ^b^	3.25 ^a^	60.430	0.627	<0.001	0.480
ADG	1.02 ^c^	1.06 ^b^	1.19 ^a^	1.06	1.12	22.400	0.040	0.149	0.356
FC	2.60 ^a^	2.91 ^b^	2.61 ^a^	2.67 ^a^	2.94 ^b^	0.054	0.019	0.007	0.491
FE	0.35 ^b^	0.35 ^c^	0.39 ^a^	0.38 ^a^	0.35 ^b^	0.007	0.018	0.007	0.656
Final weight	121.70	123.70	127.70	120.00 ^a^	128.70 ^b^	1.260	0.071	<0.001	0.625
Total period—1 to 69 days									
ADFI	2.756	2.825	2.833	2.583 ^b^	3.027 ^a^	43.600	0.527	<0.001	0.907
ADG	1.059 ^c^	1.092 ^b^	1.143 ^a^	1.045 ^b^	1.151 ^a^	13.400	0.006	<0.001	0.429
FC	2.597 ^b^	2.587 ^b^	2.479 ^a^	2.472 ^a^	2.636 ^b^	0.025	0.050	<0.001	0.627
FE	0.387 ^b^	0.387 ^b^	0.405 ^a^	0.406 ^a^	0.381 ^b^	0.004	0.043	<0.001	0.701

^1^ ADFI—average daily feed intake; ^2^ ADG—average daily gain; ^3^ FC—feed conversion, ^4^ FE—feed efficiency. Inclusion of 10 ppm of ractopamine in the finishing 2 phase. Different letters on the same line indicate a significant difference at 5% in the Tukey test.

**Table 3:** Effects of treatments on carcass characteristics.

							*p*-Value		
Variable	CONT	PC	RAC	Gilt	Barrow	SEM	Diet	Sex	Diet × Sex
n	46	47	47	71	69				
Yield %	78.77	78.86	79.24	78.59 ^b^	79.32 ^a^	0.140	0.352	0.010	0.331
^1^ BfT, mm	14.75	14.33	14.61	14.34	14.79	0.270	0.814	0.410	0.554
^2^ MD, mm	46.10	49.40	48.90	47.80	48.50	0.870	0.246	0.656	0.908
^3^ LM%	56.05	56.63	56.42	56.46	56.27	0.170	0.377	0.590	0.603

^1^ BfT—thickness of bacon; ^2^ MD—muscle depth; ^3^ LM—lean meat: (% LM = K1 − (K2 × BfT) + (K3 × MD). Different letters on the same line indicate a significant difference at 5% in the Tukey test.

**Table 4:** Effect of different treatments on meat quality (Longissimus dorsi) 24 h after slaughter.

							*p*-Value		
Variable	CONT	PC	RAC	Gilt	Barrow	SEM	Diet	Sex	Diet × Sex
n	10	10	10	15	15				
pH 45 min	7.27	7.24	7.31	7.35	7.20	0.070	0.925	0.322	0.217
pH 24 h	5.72	5.78	5.81	5.75	5.79	0.020	0.350	0.365	0.886
^1^ SF (N)	39.10 ^b^	37.00 ^c^	48.00 ^a^	42.30	40.40	1.710	0.023	0.558	0.962
^2^ CL %	23.10	22.30	23.30	23.60	22.10	0.890	0.909	0.431	0.588
^3^ DL %	2.26	1.94	2.01	2.11	2.03	0.200	0.799	0.834	0.309

^1^ SF: shear force; ^2^ CL: cooking loss, ^3^ DL: drip loss. Different letters on the same line indicate a significant difference at 5% in the Tukey test.

**Table 5:** Effects of different treatments on the shelf life of meat (Longissimus dorsi) stored after 3 days.

							*p*-Value		
Variable	CONT	PC	RAC	Gilt	Barrow	SEM	Diet	Sex	Diet × Sex
n	10	10	10	15	15				
pH 3 d	5.73	5.72	5.77	5.71	5.77	0.02	0.483	0.144	0.517
L* 24 h	51.30	51.80	50.10	49.89	52.20	0.640	0.520	0.083	0.889
a* 24 h	11.60 ^a^	10.60 ^b^	9.41 ^c^	9.98 ^b^	11.09 ^a^	0.280	0.001	0.014	0.273
b* 24 h	16.28 ^a^	15.68 ^b^	14.30 ^c^	14.86 ^b^	15.98 ^a^	0.260	0.001	0.005	0.281
Chroma value 24 h	19.89 ^a^	18,93 ^a^	16.99 ^b^	17.91 ^b^	19.43 ^a^	0.379	0.001	0.128	0.308
Hue Angle 24 h	54.67	56.01	56.89	56.28	55.38	0.379	0.053	0.206	0.377
Color L* 3 d	58.13	57.92	58.59	57.65	58.76	0.380	0.759	0.158	0.312
Color a* 3 d	12.23 ^a^	11.76 ^b^	10.59 ^c^	11.44	11.61	0.230	0.005	0.649	0.084
Color b* 3 d	14.15 ^a^	13.73 ^b^	12.52 ^c^	13.54	13.39	0.190	<0.001	0.626	0.284
Chroma values 3 d	18.68 ^a^	18.08 ^a^	16.36 ^b^	17.73	17.70	0.300	0.002	0.961	0.177
Hue Angle 3 d	49.29	49.46	49.94	49.91	49.16	0.314	0.680	0.224	0.089
^1^ TBARS μg/kg	1.67 ^c^	1.49 ^b^	1.29 ^a^	1.53	1.44	0.050	0.010	0.359	0.424

^1^ Thiobarbituric acid reactive substances. Different letters on the same line indicate a significant difference at 5% in the Tukey test.

## Data Availability

Not applicable.
